# The NbCBP1-NbSAMS1 Module Promotes Ethylene Accumulation to Enhance *Nicotiana benthamiana* Resistance to *Phytophthora parasitica* Under High Potassium Status

**DOI:** 10.3390/ijms26031384

**Published:** 2025-02-06

**Authors:** Sadia Noorin, Youwei Du, Yi Liu, Shuanghong Wang, Yan Wang, Hongchen Jia, Tom Hsiang, Rong Zhang, Guangyu Sun

**Affiliations:** 1 State Key Laboratory of Crop Stress Resistance and High-Efficiency Production and College of Plant Protection, Northwest A&F University, Yangling, Xianyang 712100, China; sadia.noorin@nwafu.edu.cn (S.N.); dyw123@nwafu.edu.cn (Y.D.); 382390039@nwafu.edu.cn (Y.L.); wsh@nwafu.edu.cn (S.W.); wy1230@nwafu.edu.cn (Y.W.); jhc@nwafu.edu.cn (H.J.); 2 School of Environmental Sciences, University of Guelph, Guelph, ON N1G 2W1, Canada; thsiang@uoguelph.ca

**Keywords:** potassium, plant disease resistance, ethylene, SAMS1, 26S proteasome

## Abstract

Potassium (K) fertilization is crucial for plant resistance to pathogens, but the underlying mechanisms remain unclear. Here, we investigate the molecular mechanism by which the addition of K promotes resistance in *Nicotiana benthamiana* to *Phytophthora parasitica*. We found that *N. benthamiana* with high K content (HK, 52.3 g/kg) produced more ethylene in response to *P. parasitica* infection, compared to *N. benthamiana* with low-K content (LK, 22.4 g/kg). An exogenous ethylene application effectively increased resistance in LK *N. benthamiana* to the level under HK status, demonstrating the involvement of ethylene in the HK-associated resistance in *N. benthamiana*. Further, transcriptome analysis showed that *NbSAMS1*, encoding ethylene biosynthesis, was induced to upregulate *P. parasitica* about five times higher in HK than in LK *N. benthamiana. NbSAMS1* overexpression enhanced resistance in LK plants, whereas *NbSAMS1* silencing reduced resistance in HK plants, confirming its importance in conferring resistance. Furthermore, we identified a calcium-binding protein, NbCBP1, which interacted with NbSAMS1, promoting its expression in HK *N. benthamiana.* Silencing *NbCBP1* compromised resistance in HK *N. benthamiana*, whereas its overexpression improved resistance in LK *N. benthamiana*. Notably, NbCBP1 protected NbSAMS1 from degradation by the 26S proteasome, thereby sustaining ethylene accumulation in HK *N. benthamiana* in response to *P. parasitica* infection. Thus, our research elucidated some mechanisms of the NbCBP1-NbSAMS1 module associated with disease resistance in HK *N. benthamiana.*

## 1. Introduction

Potassium (K) is a critical macronutrient for plant growth and development, and is essential for various physiological and biochemical processes, such as protein synthesis, osmoregulation, ionic balance, and enzyme activation. K also emerges as a positive regulator of plant defense. Numerous studies have demonstrated the protective benefits of K fertilizer application against diverse plant pathogens [1]. Sufficient K or high K (HK) levels have been associated with enhanced resistance to pathogens, while insufficient K can compromise plant immunity [2]. Optimal potassium levels vary across different plants. For *N. benthamiana*, the optimum foliar potassium content is 4.0%, which is the lower limit of the optimal potassium level for tobacco [3]. Improper potassium fertilization can affect various aspects of plant health, soil quality, and the environment. In areas where the average K content of foliage is well below optimum [4,5,6], these deficiencies have contributed to an increased incidence of apple Valsa canker. However, applications of potassium causing tissue to exceed recommended levels may lead to significant decreases in yields, and may even result in nutrient loss due to excessive leaching [7]. In field settings, the application of K fertilizer has been shown to reduce the impact of *Cytospora mali* damage on apples [8]. The plant hormone, ethylene, is a key regulator in various aspects of plant processes, especially as a defense response to pathogen infection [9]. Exogenous ethephon could protect plants against the necrotrophic fungus *Botrytis cinerea* [10]. Ethylene accumulation has also been reported to enhance plant resilience to various abiotic stresses [11,12]. Thus, an understanding of the relationship between K supply and plant resistance can help us design agricultural strategies that support crop nutrition and enhanced resilience [13].

The S-adenosyl-l-methionine synthetase gene (*SAMS*) plays a pivotal role in ethylene biosynthesis. SAMS also contributes to plant development and stress responses [14,15]. For example, CLCuMuV C4 suppresses transcriptional and post-transcriptional gene silencing-based antiviral defense in cotton by targeting and inhibits *NbSAMS2* [14]. PlAvh202 promotes the degradation of LcSAMS through 26S proteasome-mediated pathways, reducing LcSAMS-catalyzed ethylene production and weakening plant resistance to *Peronophythora litchi* [16]. Thus, SAMS catalyzed ethylene synthesis may be a potential key process in plant resistance to pathogens.

Plants have evolved multi-level defense mechanisms to combat pathogen infections [17]. Calcium signaling serves as a secondary messenger in signal transduction, regulating physiological and biochemical processes in plants and improving plant resistance [18]. Plant calcium-binding proteins include calmodulin (CaM), calmodulin-like (CML), calcium-dependent protein kinase (CDPK), and calcineurin B-like proteins (CBL) [19]. These proteins recognize calcium signals and trigger defense responses, such as defense genes activations, ROS burst, and the production of nitric oxide, and plant hormones such as salicylic acid, jasmonic acid, and ethylene [20]. Numerous studies have found that potassium nutrition is closely related to the expression of *CML* genes [21,22]. The increase in intracellular calcium ion concentration coincides with rapid membrane depolarization and potassium efflux during the initial phases of pathogen response [23]. Thus, these results imply that there may be a close relationship between calcium-binding proteins and potassium in activation of plant disease.

In this study, we found that ethylene accumulation plays an essential role in the resistance of *N. benthamiana* to *P. parasitica*. Notably, we found that a calcium signaling pathway protein, NbCBP1, interacts with and stabilizes NbSAMS1 by blocking 26S proteasome ubiquitination/degradation, thereby sustaining ethylene accumulation under HK and during pathogen infection. Overall, we found that the NbCBP1-NbSAMS1 module promoted ethylene accumulation and was responsible for enhanced resistance in HK *N. benthamiana.*

## 2. Results

### 2.1. High Potassium Levels Promote Ethylene Accumulation After Inoculation

**Figure 1:**
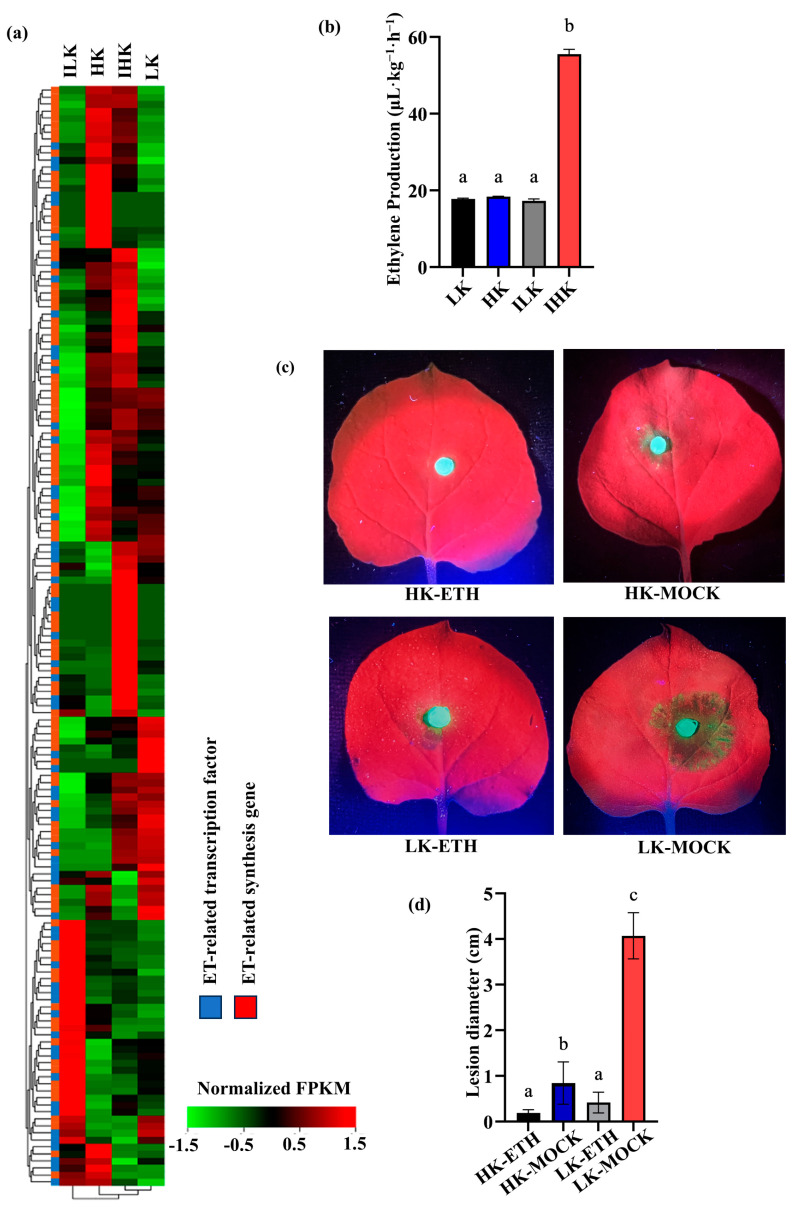
Ethylene is important in enhancing disease resistance. (**a**) Heatmap displays the expression variation in ethylene-synthesis and associated transcription factors in plants with high potassium content (HK), low potassium (LK), *P. parasitica*-infected HK (IHK), and *P. parasitica*-infected LK (ILK) shown in red and green. The scale bar represents the normalized FPKM of each gene. (**b**) Ethylene production in HK, LK, IHK, and ILK *N. benthamiana*. (**c**) Representative images and (**d**) comparative analysis of lesion diameter in HK plants treated with ethephon (HK-ETH), LK plants treated with ethephon (LK-ETH), HK plants without ethephon treatment (HK-MOCK), or LK plants without ethephon treatment (LK-MOCK). Data are shown as means ± SD and different letters represent significant differences among treatments in lesion diameter at *p* < 0.05 using Student’s *t*-tests.

We grew *N. benthamiana* under low potassium and high potassium conditions for three weeks to obtain plants with low potassium (LK) and high potassium (HK) contents (Table S1). The leaf K assessment showed that the K content in HK *N. benthamiana* was 52.3 g/kg, which was significantly higher than that of LK (22.6 g/kg) (Table S1). And there were no significant differences in the levels of nitrogen (N) and phosphorus (P) among all HK and LK plants (Table S1). Typically, this high planta K content was the optimal or standard level for both resistance responses and the primary growth of *N. benthamiana* [24]. Further transcriptomic analysis revealed that the expressions of genes involved in ethylene (ET) synthesis and signaling were more active in HK *N. benthamiana* infected by *P. parasitica* (IHK), than in infected LK *N. benthamiana* (ILK) (Figure 1a). Among 158 ET-related genes, the expression levels of 120 were higher in IHK *N. benthamiana* than those in ILK (Figure 1a), indicating the involvement of ethylene-related processes in the high resistance of HK *N. benthamiana* to *P. parasitica*.

Ethylene production was measured in *N. benthamiana* leaves on a fresh-weight basis. No significant differences were observed in ethylene levels between non-inoculated LK and HK plants (Figure 1b). However, upon *P. parasitica* inoculation, a significant increase in ethylene production was detected in IHK plants (55.52 μL·kg^−1^·h^−1^) compared to ILK plants (17.25 μL·kg^−1^·h^−1^) (Figure 1b). This indicated that HK plants produced higher levels of ethylene in response to pathogen infection.

To further assess the role of ethylene in plant disease resistance, the effect of exogenous ethephon (ETH) was explored. At 36 h post-inoculation (hpi), high potassium plants treated with ethephon (HK-ETH) 24 h earlier, exhibited no lesions on leaves, similar to low potassium plants with ethephon treatment (LK-ETH), which showed reduced lesions diameter (0.4 ± 0.2 cm). High potassium plants without ethephon treatment (HK-MOCK) developed small lesions (0.8 ± 0.2 cm) on leaves, while low potassium plants without ethylene treatment (LK-MOCK) showed significantly larger lesions (4.1 ± 0.2 cm) (Figure 1c,d). The results revealed that ethylene-treated plants exhibited high resistance to *P. parasitica*, even in LK *N. benthamiana*, demonstrating that ethylene-enhanced *N. benthamiana* resistance to *P. parasitica* was related to K status.

### 2.2. NbSAMS1 Positively Regulates Nicotiana benthamiana Resistance to Phytophthora parasitica

**Figure 2:**
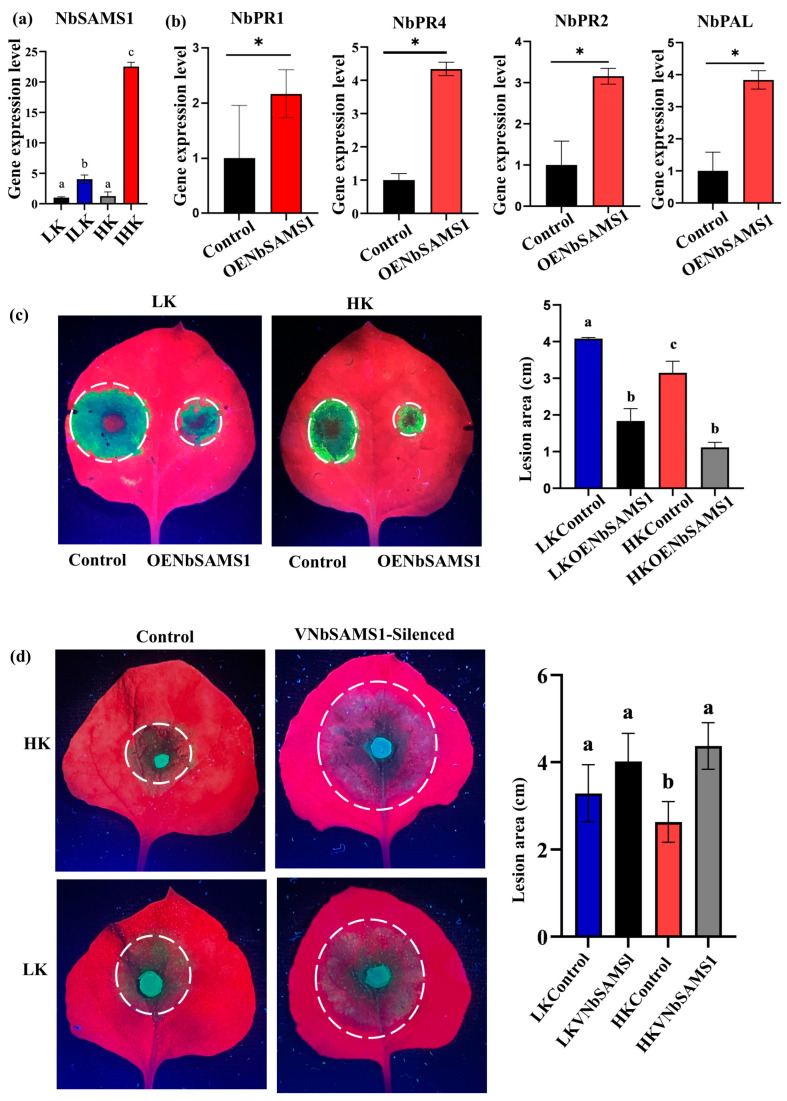
*NbSAMS1* plays a positive role in enhancing disease resistance in *Nicotiana benthamiana*. (**a**) *NbSAMS1* expression in LK, HK, ILK, and IHK *N. benthamiana.* (**b**) Upregulation of PR genes in overexpressed *NbSAMS1* in *N. benthamiana* inoculated with *P. parasitica*. (**c**) Lesion diameter analyses of *NbSAMS1* overexpressing (OENbSAMS1) HK and LK *N. benthamiana* leaves (**d**) Lesion diameter analyses of *NbSAMS1* silenced (VNbSAMS1) HK and LK *N. benthamiana* leaves. Data are shown as means ± SD using Student’s *t*-test analysis. *: *p* < 0.05; different letters denote significant differences at *p* < 0.05.

*SAMS1* plays a pivotal role in ethylene biosynthesis, which is critical for plant defense. Previous research has also demonstrated its role in plant development and stress responses [14,15]. To understand the relationships between K contents in plants and ethylene synthesis, the expression of *NbSAMS1* in HK and LK *N. benthamiana* was analyzed. No significant differences in *NbSAMS1* expression were observed between non-inoclated HK and LK plants. However, *P. parasitica* inoculation significantly upregulated *NbSAMS1* expression in HK *N. benthamiana*, which was about five times higher than that in LK (Figure 2a). This indicated that pathogens could enhance the expression of *NbSAMS1*, especially in plants with HK status. We also found that the expression level of pathogenesis-related (PR) genes *NbPR1*, *NbPR4*, *NbPR2*, and *NbPAL* were significantly upregulated compared to the control (Figure 2b), implying that NbSAMS1 may act as a positive regulator of plant immunity.

To investigate the role of *NbSAMS1* in plant resistance, we constructed overexpression (OENbSAMS1) and silencing (VNbSAMS1) vectors and generated transgenic plants. RT-qPCR confirmed increased overexpression of *NbSAMS1* in both HK and LK *N. benthamiana* (Figure S1a); however, NbSAMS1 expression was reduced to 60% in silenced *N. benthamiana* (Figure S1b). A pathogenicity assay revealed that *NbSAMS1* overexpression significantly reduced the lesion size in *N. benthamiana* even with LK status, which was smaller than that of the non-overexpressed IHK control (Figure 2c). The lesion diameter after *NbSAMS1* silencing was significantly enlarged in *N. benthamiana* with HK status (Figure 2d). These results showed that *NbSAMS1* was a positive regulator of *N. benthamiana* resistance to *P. parasitica.*

### 2.3. NbSAMS1 Interacts Directly with NbCBP1

**Figure 3:**
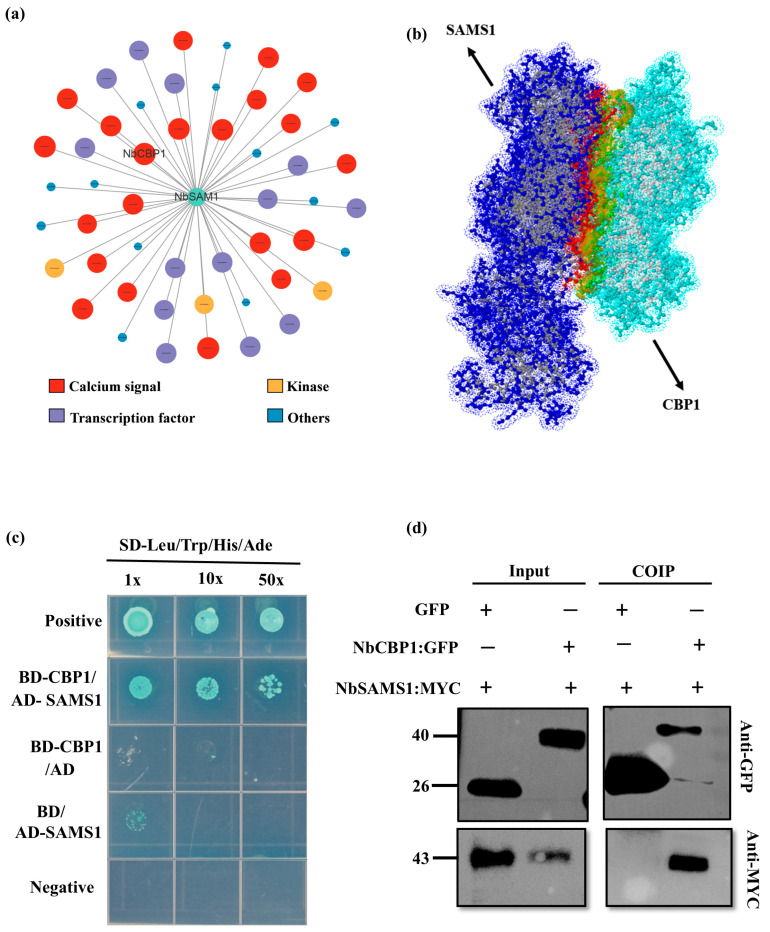
*NbCBP1* interacts with *NbSAMS1*. (**a**) Coexpression network of *NbCBP1* and *NbSAMS1* with genes involved in the hub module. (**b**) Molecular docking analysis of NbSAMS1 and NbCBP1 interaction. (**c**) Yeast two-hybrid assay displays the interaction between NbCBP1 and NbSAMS1. (**d**) Co-immunoprecipitation (Co-IP) assay confirms the NbCBP1-NbSAMS1 interaction in vivo.

We constructed a hub network of *NbSAMS1* and *NbCBP1* expression along with their related genes to better understand their functional roles in plant defense. The analysis showed a strong correlation between *NbCBP1* and *NbSAMS1*, suggesting their interconnected roles in plant resistance. Additionally, these genes were associated with the expression of other genes involved in key defense mechanisms, such as transcriptional regulation, calcium signaling transduction, and kinase activity, further underscoring the importance of *NbCBP1* and *NbSAMS1* in plant defense. The predicted network showed that *NbCBP1* might act as an upstream regulatory protein of *NbSAMS1* (Figure 3a). This was further supported by molecular docking analysis, which indicated a strong interaction between the two proteins, with a binding energy of ΔiG = −16.1 kcal/mol (Figure 3b, Table S4). To confirm this interaction, we used a yeast two-hybrid (Y2H) assay (Figure 3c). The results indicated direct interaction between NbCBP1 and NbSAMS1. Then, the interaction between NbCBP1 and NbSAMS1 was confirmed by CoIP in vivo (Figure 3d).

### 2.4. NbCBP1 Affects Ethylene Accumulation and Positively Regulates Nicotiana benthamiana Resistance

**Figure 4:**
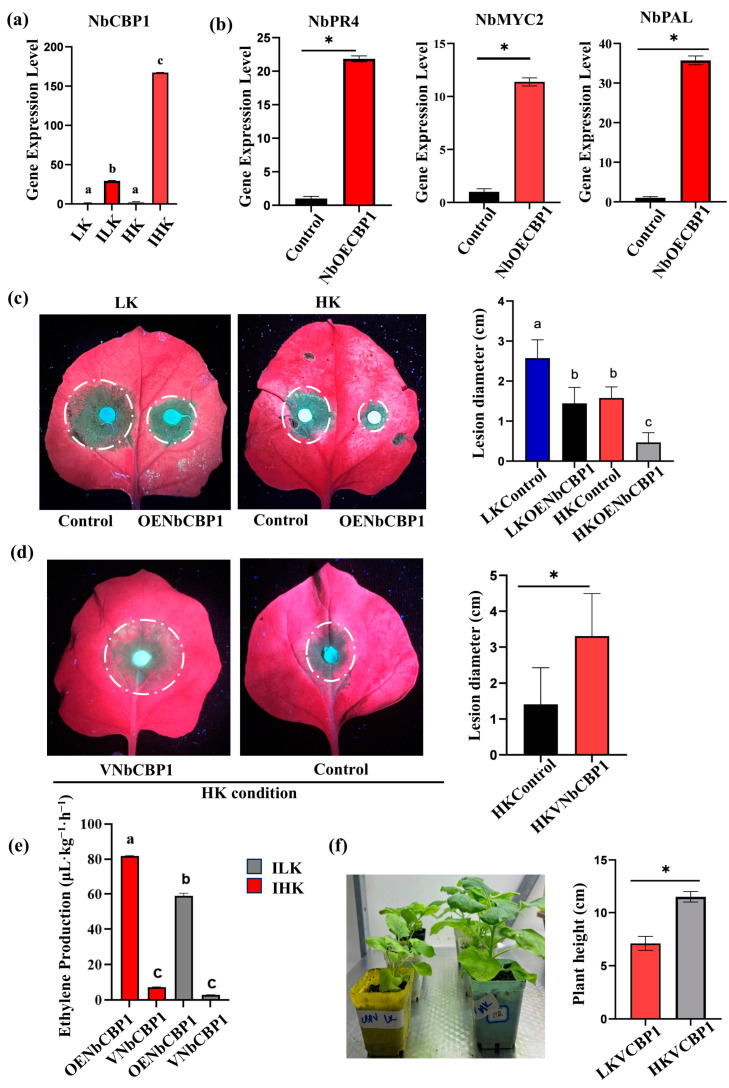
*NbCBP1* enhances *Nicotiana benthamiana* resistance under high potassium conditions. (**a**) *NbCBP1* expression in HK, LK, IHK, and ILK *N. benthamiana.* (**b**) Upregulation of PR genes in overexpressed *NbCBP1* in *N. benthamiana* inoculated with *P. parasitica.* (**c**) Lesion diameter analyses of *N. benthamiana* leaves (HK or LK) with *NbCBP1* overexpressing. (**d**) Lesion diameter analyses of *NbCBP1* silenced HK *N. benthamiana* leaves. (**e**) Ethylene production in *NbCBP1* overexpression and silenced IHK and ILK *N. benthamiana*. (**f**) Plant height of *NbCBP1* silenced LK and HK *N. benthamiana.* Data are shown as means ± SD, *: *p* < 0.05; different letters denote significant differences at *p* < 0.05 from Student’s *t*-test.

To investigate whether *NbCBP1* is related to K levels, the expression of *NbCBP1* was assessed in HK and LK *N. benthamiana.* The results showed that *NbCBP1* was significantly induced in HK and LK *N. benthamiana* following *P. parasitica* challenge. The expression of *NbCBP1* was much higher, about six times in IHK than in ILK *N. benthamiana.* However, there were no differences in *NBCBP1* expression between HK and LK *N. benthamiana*. These results showed that *NbCBP1* could be differentially induced by pathogen infection and that K content could promote its upregulation (Figure 4a). To assess the impact of *NbCBP1* on plant resistance, we analyzed the transcription levels of PR genes, and found that the expression of *NbMYC2*, *NbPAL*, and *NbPR4* was significantly upregulated (Figure 4b), implying that *NbCBP1* may act as a positive regulator of plant immunity.

To assess whether *NbCBP1* affects plant disease resistance, we overexpressed *NbCBP1* in *N. benthamiana.* RT-qPCR analysis confirmed *NbCBP1* overexpression in both HK and LK *N. benthamiana* (Figure S2a). Then, we inoculated the leaves with *P. parasitica*, and at 3 dpi, we found that the lesions on LK or HK *N. benthamiana* overexpressing *NbCBP1* were significantly smaller than those of the corresponding non-overexpressed control, indicating that *NbCBP1* could enhance host immunity (Figure 4c). We further investigate the function of *NbCBP1* in *N. benthamiana* using the VIGS system. After infiltration, the efficiency of gene silencing was verified by RT-qPCR. The results showed that the expression levels of *NbCBP1* in both LK and HK *N. benthamiana* were significantly suppressed (Figure S2b). Pathogenicity assays also showed that the lesion diameters on HK *N. benthamiana* leaves with *NbCBP1* silencing were larger than that in HK controls (Figure 4d). In parallel, *NbCBP1* silencing did not affect lesion development in LK leaves (Figure S2c). These results supported the positive regulatory effect of *NbCBP1* on plant disease resistance.

Next, we investigated the role of *NbCBP1* in ethylene production in *N. benthamiana*. Ethylene accumulation was significantly higher in the *NbCBP1*-overexpressing IHK plants, which produced 81.8 μL·kg^−1^·h^−1^, compared to the *NbCBP1*-overexpressing ILK plants, which had 58.9 μL·kg^−1^·h^−1^. Ethylene production was considerably lower in the *NbCBP1*-silenced plants, with the VNbCBP1-IHK plants showing 7.2 μL·kg^−1^·h^−1^ and the VNbCBP1-ILK plants showing 2.9 μL·kg^−1^·h^−1^ (Figure 4e). These silenced plants had significantly lower ethylene production, but they were not different from each other (Figure 4e). Additionally, we also observed that silencing *NbCBP1* affected plant height, with LK *N. benthamiana* showing a height of 7.1 cm compared to 11.5 cm in HK *N. benthamiana* (Figure 4f). These findings indicated that *NbCBP1* played a crucial role in ethylene synthesis and could be upregulated by pathogen infection, especially in HK plants.

### 2.5. NbCBP1 Blocks Ubiquitination/Degradation to NbSAMS1 to Promote Ethylene Accumulation in HK N. benthamiana

**Figure 5:**
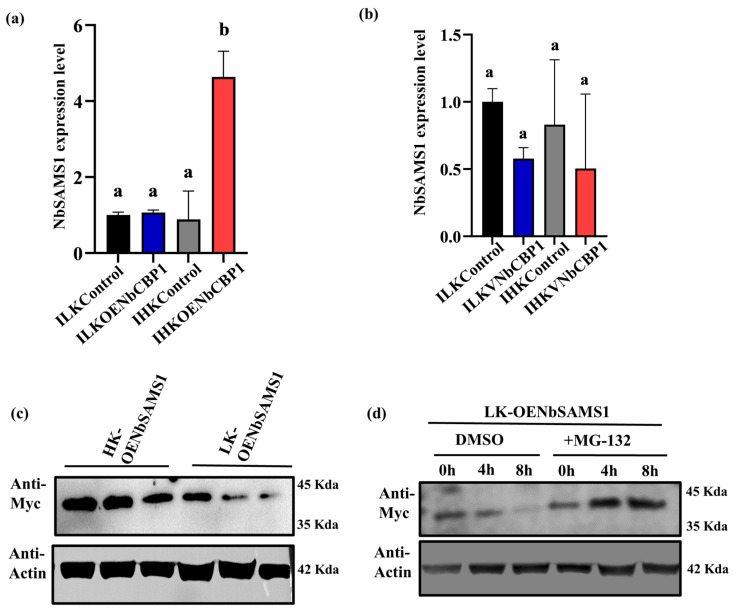
NbCBP1 stabilizes NbSAMS1 and promotes ethylene accumulation in response to K availability and pathogen stress. Expression of *NbSAMS1* in ILK and IHK *N. benthamiana* with (**a**) *NbCBP1* overexpression or (**b**) *NbCBP1* silencing. (**c**) Immunoblot analysis in *NbSAMS1* overexpressing HK and LK *N. benthamiana*. (**d**) NbSAMS1 degradation assays performed with or without MG132 treatment in LK *N. benthamiana.* Data are shown as means ± SD; different letters denote significant differences at *p* < 0.05 from Student’s *t*-test.

The predicted interaction network showed that CBP1 might be an upstream regulatory protein of SAMS1 (Figure 3a). We then overexpressed *NbCBP1* and quantitatively measured the expression of *NbSAMS1,* and found no significant difference in *NbSAMS1* expression in *N. benthamiana* among LK, HK, and ILK plants. However, *NbSAMS1* expression significantly increased in IHK plants (Figure 5a). After silencing *NbCBP1*, there were no differences in *NbSAMS1* expression in IHK plants compared with LK, HK, and ILK plants (Figure 5b). These results suggested that NbCBP1 is an upstream regulation protein of NbSAMS1, and the high expression of *NbSAMS1* in IHK *N. benthamiana* was induced by *NbCBP1*.

Previous studies have shown that *SAMS1* undergoes protein degradation influenced by ubiquitination in several plants. For example, *OsFBK12* (a subunit of E3 ligase) targets *OsSAMS1* for degradation in rice [25]. To understand whether *NbSAMS1* is affected by ubiquitination, we used AlphaFold3 to predict the potential interactions among NbCBP1, the E3 ligase, and NbSAMS1. The results showed that there may be a direct interaction between NbCBP1 and NbSAMS1, as well as between NbSAMS1 and E3 ligase (Figure S3a,b), thus implying that the E3 ligase may mediate the degradation of SAMS1. The prediction of the interaction among these three proteins suggested that NbCBP1 exhibits a strong binding affinity for SAMS1 (Figure S3c). This suggests a competitive relationship between CBP1 and the E3 ligase for binding to NbSAMS1. Thus, we hypothesize that NbCBP1 may prevent the ubiquitination of E3 ligase and subsequent degradation of NbSAMS1.

To test the above hypothesis, we performed a Western blot to compare the molecular mass patterns of NbSAMS1. The result demonstrated that bands with higher molecular mass were found in the *HK N. benthamiana* leaves with NbSAMS1 overexpression; however, lower molecular mass bands were found in LK leaves (Figure 5c). To understand the differences caused by 26S proteasome ubiquitination, *LK N. benthamiana* leaves expressing *NbSAMS1* were treated with 26S proteasome inhibitor MG132. We found that NbSAMS1 was stable in the presence of MG132 in LK status (Figure 5d), whereas in the DMSO (control), NbSAMS1 showed a gradual decrease. These findings suggested that NbCBP1 might protect NbSAMS1 from ubiquitination/degradation by the 26S proteasome under HK status.

## 3. Discussion

Ethylene is one of the six major plant hormones, which are important regulators in plant growth, development, and reproduction. Ethylene has also been found to play an important role in plant disease resistance. The synthesis of ethylene is affected by a variety of mineral elements, such as N, P, Ca, Fe, Mn, Cu, S, Co, B, Mg, and Cd [26,27], and by pathogens. Ca availability has been shown to enhance plant disease immunity by promoting ethylene production. In mung beans, increased Ca significantly boosted ethylene production [26,28]. Many studies have shown that ethylene production is induced by infection with pathogens. For instance, ethylene is induced in tobacco plants infected with *Phytophthora parasitica* [29], in rice infected with *Magnaporthe oryzae* [30], and in carrots infected with *Botrytis cinerea* [31]. PTI and ETI utilize the PICI1-OsMETS-ethylene cascade, and ETI-PTI integration enhances rice broad-spectrum blast resistance [32]. *SAMS1* is a key gene in ethylene biosynthesis and plays a crucial role in plant disease resistance, as its expression is induced by various biotic stresses, conferring increased tolerance [33,34]. In litchi, *SAMS1* positively regulates ethylene biosynthesis and plant immunity against *Peronophythora litchii* [16]. Silencing the *SAMS* enhances plant susceptibility to CLCuMuV infection in *N. benthamiana* [14]. These studies show that ethylene plays a significant role as a modulator of plant disease resistance. Consistent with these findings, our results showed that both the high expressions of the *NbSAMS1* and the increased ethylene production all contribute to the resistance enhancement of *N. benthamiana* to *P. parasitica.* Previous studies showed that potassium deprivation induces ethylene production [35], and that adequate potassium nutrition suppresses ethylene evolution [36]. Our results showed that for ethylene evolution in *N. benthamiana,* there were no differences between high-potassium and low-potassium conditions. Unexpectedly, we found that the *P. parasitica* infection significantly promoted ethylene accumulation in HK *N. benthamiana*, which was about three times higher than that in LK *N. benthamiana.* Our findings demonstrated that high potassium or adequate potassium status could promote ethylene synthesis and improve plant resistance under biotic stress.

PR genes are involved in plant defense by activating SAR and ISR via the salicylic acid (SA), jasmonic acid (JA) or ethylene signaling pathways [37]. Additionally, *NbPAL*, involved in the phenylpropanoid pathway, promotes the production of phenolic compounds such as lignins, which reinforce physical barriers and boost immune responses against pathogens [38]. Our findings showed that *NbPR1*, *NbPR2*, *NbPR4*, and *NbPAL* were upregulated in *NbSAMS1* overexpressed plants, indicating the pivotal roles of PR-related genes in enhancing *N. benthamiana* resistance, and supporting their involvement in plant immunity pathways.

The calcium signaling pathway significantly affects plant immunity, and various calcium-binding proteins have been shown to be influenced by potassium nutrition. For example, potassium nutrition can affect calcium signaling, e.g., the calcium sensor protein genes *OsCML1*, *OsCML18*, *OsCML20*, and *OsCML31* are up-regulated in rice during low-K stress [39]. The Ca^2+^-CaM signaling pathway promotes K absorption under K stress [40]. The calcium signaling was reported to be related to ethylene signaling. Following infection, *TaCML36* expression increases in the ethylene signaling pathway, resulting in increased resistance to sharp eyespot [41]. Our study identified the novel CML family protein *NbCBP1* and its expressions in *N. benthamiana* were differentially induced in plants with varying potassium status by pathogen infection. High potassium *N. benthamiana* with pathogen challenge can promote ethylene accumulation and resistance to *P. parasitica.* These findings suggested that potassium affected plant immunity via calcium and ethylene signaling pathways. We also found that overexpression could enhance plant resistance even in LK plants, implying that *CBP1* could be potential resistance gene for developing transgenic plants. To our knowledge, this is the first report on *CBP1* involvement in promoting ethylene accumulation and plant immunity.

**Figure 6:**
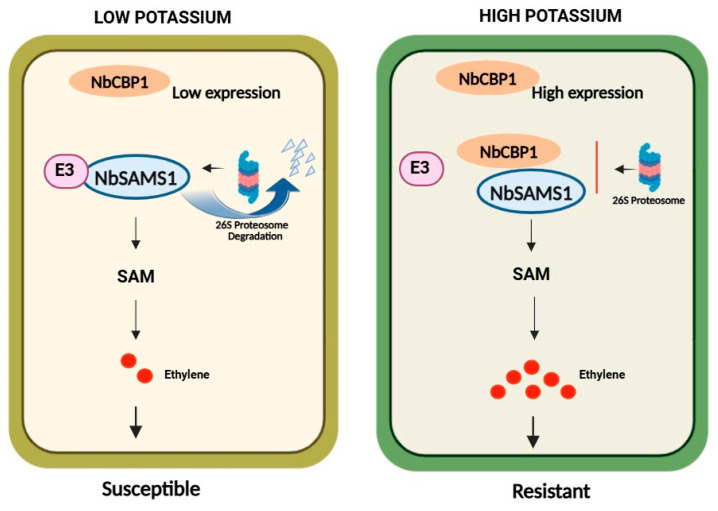
Proposed Mechanism of high resistance in *Nicotiana benthamiana* with high potassium status. In low potassium *N. benthamiana* (left), the *NbCBP1* shows low expression, leading to E3 ligase and SAMS1 interaction. SAMS1 is then degraded via 26S proteasome. This degradation results in lower ethylene production, weakening the plant’s defense response. In high potassium *N. benthamiana* (right), the *NbCBP1* shows high expression; CBP1 competes with E3 ligase to interact with NbSAMS1, thereby protecting SAMS1 from degradation, enhancing the stability of SAMS1, resulting in increased ethylene production. The elevated ethylene levels contribute to the plant’s enhanced plant disease resistance.

As a major macro-nutrient, potassium content profoundly affects disease severity [13]. Earlier research has shown that sufficient potassium supply reduces lipid peroxidation and preserves lipid homeostasis, thereby mitigating the adverse effects of sheath rot caused by *Sarocladium oryzae* on rice [42]. Sufficient potassium supply (0.93%) in apple branches enhances resistance to *Cytospora mali* through the promotion of several antifungal secondary metabolites, especially sufficient coumarin accumulation [43]. Apart from the accumulation of reactive oxygen species and antifungal metabolites, the nucleotide-binding domain and leucine-rich repeat resistance genes (NLR) are activated in high-potassium *N. benthamiana* [24]. These research findings show that the mechanisms of K-enhanced plant immunity must be highly complicated. This study found that high potassium status enhanced plant immunity by promoting ethylene accumulation. Furthermore, the key calcium-binding protein NbCBP1 was identified. On the one hand, overexpression of *NbCBP1* in *N. benthamiana* promoted the high expression of *NbSAMS1*, and silencing *NbCBP1* in *N. benthamiana* led to a lower expression of *NbSAMS1*, implying that NbCBP1 caused high expression of *NbSAMS1* in HK plants upon pathogen infection. On the other hand, we found that NbCBP1 interacted with NbSAMS1 and protected it from ubiquitination/degradation in HK plants, which was particularly important after pathogen infection. Taken together, these results supported the hypothesis that upon pathogen infection, *NbCBP1* was induced and highly expressed in high potassium plants, which promoted high *NbSAMS1* expression. NbCBP1 further interacted with NbSAMS1, protecting it from ubiquitination degradation, keeping NbSAMS1 at a high level, and enhancing ethylene accumulation and plant disease resistance (Figure 6). Our results support the role of NbCBP1 in stabilizing NbSAMS1 via potential E3 ligase ubiquitination. Future research will focus on identifying the details of ubiquitination proteins involved in the ubiquitination of NbSAMS1. Several observations indicate that applying ethylene before infection with pathogens reduces the disease severity [44]. Our results showed that exogenous ethephon treatment on LK plants significantly increased plant disease resistance, suggesting ethylene functions in increasing plant immunity. The exogenous ethylene application activates resistance responses against a broad spectrum of pathogens. This could be used as an additional strategy to protect plants from diseases in low potassium fields.

In conclusion, we uncovered a novel mechanism by which K influences ethylene accumulation and plant immunity. This mechanism is critical for enhancing plant pathogen resistance and highlights a significant dimension of the role of potassium in plant defense.

## 4. Materials and Methods

### 4.1. Plant Materials

*N. benthamiana* seedlings were transplanted into plastic pots with sterile mixtures of vermiculite and soil substrate at 1:1 (*v*/*v*) [45]. Subsequently, the seedlings were grown in a modified Hoagland’s nutrient solution (Table S2). To achieve high-K plants (4% to 6%) [46], the solution contained two-fold the amount of KNO_3_ (0.758 g/L). Conversely, to obtain low-K plants (<2%), the solution had no KNO_3_ (0 g/L). To ensure an equal nitrogen level in both solutions, 0.3 g/L NH_4_NO_3_ was added into the 0 K nutrient solution. After three weeks of incubation in the growth chamber at 23 °C with a 16 h light and 8 h dark photo period and ~60% humidity, the N, P, and K content in the dry leaf weight were measured, and the HK and LK *N. benthamiana* leaves were harvested for the further experiment. There were three replicates for each treatment, and the entire experiment had three repetitions.

### 4.2. Pathogen Inoculation and Disease Severity

The *Phytophthora parasitica var. nicotianae* strain was grown on PDA at 25 °C. The strain was provided by Prof. Xili Liu (Northwest A&F University), and it was used in previous research to infect *N. benthamiana* [43]. Hyphal plugs with a 55 mm diameter of *P. parasitica* were inoculated into nonwounded detached leaves from *N. benthamiana* seedlings. At 3 dpi, the inoculum plugs were removed, and representative leaves were photographed for evaluation. Each inoculation experiment had at least three replicates, and the experiment was conducted three times.

### 4.3. Construction of Vectors and Genetic Transformation in N. benthamiana

TRV vectors were constructed by cloning 350 bp DNA segments of *NbSAMS1* (Ni-ben101Scf04643g02010.1) and its target gene *NbCBP1* (Niben101Scf08035g00009) of *N. benthamiana* using a primer pair containing *Eco*RI and *Bam*HI sites and then ligating them to pTRV2 (Table S3). Appropriate corresponding recombinant vectors were introduced into *Agrobacterium* GV3101, which was then used to transform both LK and HK *N. benthamiana* as previously described [47]. The control group consisted of an Agrobacterium solution with an empty vector. Silenced *N. benthamiana* leaves were subsequently inoculated with *P. parasitica* as above. RT-qPCR was used to analyze *NbCBP1* and *NbSAMS1* transcript levels in plants at 3 days post-incubation. The full-length cDNA of *NbCBP1* (Niben101Scf08035g00009) was amplified using a primer pair containing BamHI sites and then cloned into the pBIN-eGFP overexpression vector, and full-length cDNA of *NbSAMS1* (Niben101Scf04643g02010.1) was amplified using a primer pair containing BamHI sites and then cloned into the MYC overexpression vector (Table S3). The overexpressing *N. benthamiana* was inoculated with *P. parasitica* and analyzed following agro-infiltration using RT-qPCR, as previously described in [47]. There were three replicates of each treatment, and the entire experiment had three repetitions.

### 4.4. Total RNA Extraction and RT-qPCR Analysis

The Omega Plant RNA Kit (Omega Bio-Tek) was used to extract the total RNA from *N. benthamiana* leaves and then Easy Script cDNA Synthesis Kit (Transgene, Beijing, China) was then used to reverse-transcribe the whole RNA to cDNA. Gene-specific primers and SYBR Green staining were used to measure the levels of gene expression in triplicate. For RNA samples, the expression of *NbEF1a* served as an internal standard. We extracted and reverse-transcribed the RNA to cDNA from pathogen-inoculated *N. benthamiana* leaves with HK and LK status. Using these gene-specific primers, RT-qPCR was used to measure the gene expression with three biological replicates for each treatment, and three technical replicates per biological sample. Gene-specific primers were synthesized based on the cDNA sequences (Table S3). The 2^−△△CT^ technique was used to analyze the RT-qPCR data.

### 4.5. Protein Extraction and Western Blotting

Agroinfiltrated HK and LK *N. benthamiana* leaves were collected and ground in liquid nitrogen. Total proteins were extracted with RIPA extraction buffer (17). After adding the extracted and purified recombinant proteins to the loading buffer sodium dodecyl sulfate–polyacrylamide gel electrophoresis (SDS-PAGE), they were boiled and centrifuged. Proteins were transferred to polyvinylidene difluoride membranes after the supernatants were obtained from different treatments, and then analyzed by PAGE. The membranes were incubated using the appropriate antibodies for the detection of NbSAMS1 and NbCBP1 protein [48].

### 4.6. IP-MS Analysis for Target Gene Identification

For IP-MS assays, the recombinant plasmid of NbSAMS1 was fused with the MYC tag to acquire the NbSAMS1-MYC protein. The MYC fused protein was overexpressed in *N. benthamiana* leaves. The supernatant (about 1 mL) of total proteins was extracted from both HK and LK *N. benthamiana* leaves and combined with magnetic beads that contained the GFP antibody at 4 °C overnight by a vertical mixer. The beads were then collected, washed six times with an IP buffer, and finally suspended beads in 120 μL IP buffer. The protein complex was assessed by immunoblotting using anti-MYC antibody to obtain candidate interactants.

For mass spectrometry analysis, the lyophilized protein samples were dissolved using 10 μL of solution and then centrifuged at 14,000× *g* at room temperature for 20 min to remove debris. The mobile phases of liquid chromatography consisted of Phases A that contained 100% water, 0.1% formic acid and Phase B contained 80% acetonitrile, 0.1% formic acid. A total of 1 μg of the supernatant was used for detection. The analysis was conducted using the EASY-LC™ 1200 UHPLC system (Thermo Fisher, Waltham, MA, USA) connected to a Q Exactive HF-X mass spectrometer (Thermo Fisher, Waltham, MA, USA) operating in the data-dependent acquisition (DDA) mode. A homemade C18 Nano-Trap column (2 cm × 75 μm, 3 μm) was used to trap the peptide from the sample. Using linear gradient elution, peptides were separated on a handmade analytical column (15 cm × 150 μm, 1.9 μm). Peptide were ionized using Nanospray FlexTM (Thermo Fisher, Waltham, MA, USA) (ESI) with spray voltage of 2.3 kV, and an ion transport capillary temperature was set at 320 °C to facilitate efficient ionization. The isolated peptides were examined using Q Exactive HF-X (Thermo Fisher, Waltham, MA, USA). The mass spectrometer was set to a resolution of 120,000 at *m*/*z* 200 for the full screen which covered the *m*/*z* from 350 to 1500, and an automated gain control (AGC) target was set of 3 × 106. The maximum ion injection period was 80 ms. The higher energy collisional dissociation (HCD) was used to select and fragment the 30 most abundant precursor ions from the entire scan. These were then subjected to MS/MS analysis with a resolution of 15,000 (at *m*/*z* 200), an automatic gain control (AGC) target value of 5 × 106, a maximum ion injection time of 100 ms, a normalized collision energy of 27%, an intensity threshold of 5 × 103, and a dynamic exclusion parameter of 20 s. The raw data obtained from the mass spectrometer were analyzed to identify potential interacting proteins.

### 4.7. Co-IP Assays

The Co-IP assay was conducted using the procedure outlined in [17]. The recombinant plasmid of NbCBP1 was fused with the GFP tag for acquiring NbCBP1-GFP proteins, while the NbSAMS1 were fused with the MYC tag for acquiring NbSAMS1-MYC proteins, respectively. GFP and MYC fusion proteins were co-expressed transiently in the leaves of *N. benthamiana.* The supernatant (approximately 1 mL) of total proteins was extracted from *N. benthamiana* leaves and combined with magnetic beads containing the GFP antibody using a vertical mixer at 4 °C overnight. The beads were collected, washed six times with an IP buffer, and then resuspended in 120 μL IP buffer. The protein complex was detected by immunoblotting using anti-GFP and anti-MYC to detect interaction. The experiment was repeated 3 times.

### 4.8. Y2H Assay

The Y2H assays were conducted using the ProQuest Two-Hybrid System (Invitrogen, Carlsbad, CA, USA). The protein-coding regions of each target were inserted in-frame into the pGADT7 vector to function as the bait. The open reading frames of the intracellular C-terminal segments of *NbCBP1* were inserted into the pGBKT7 vector to be used as prey (Table S3). Positive clones were identified after the co-transformation of yeast (Y2H gold strain) and screening on SD/Leu-Trp-His plates. They were then grown on SD/Leu-Trp-His-Ade medium with varied concentrations of ABA and X-α-gal or assayed with β-galactosidase (X-gal). The experiment was conducted three times.

### 4.9. Exogenous Etephon Treatment

For the exogenous ethylene treatment, a solution was prepared by mixing 499 mL of distilled water, 1 mL of 40% ethephon (a plant growth regulator that releases ethylene), and 150 µL of organic silicon surfactant. The final concentration of ethephon in the solution was 0.08%. The prepared solution was thoroughly mixed and sprayed uniformly onto the leaves of *N. benthamiana* plants. The plants were left undisturbed for 24 h to allow the solution to be absorbed. The treatment conditions were kept consistent for the experimental groups, which included HK and LK *N. benthamiana*. Each treatment was repeated three times, and the entire experiment was conducted in triplicate.

### 4.10. Quantification of Ethylene

*N. benthamiana* leaves were treated with agroinfiltration, and after 24 h they were detached and weighed. Leaves were sealed in a 10 mL glass vial at 25 °C for 6 h. To quantify ethylene concentration, a 1 μL gas sample from the head space was injected into the gas chromatography (Agilent 7890B, Santa Clara, CA, USA) using a gas-tight syringe (Hamilton, Reno, NV, USA). The column (Agilent, GS-Alumina; 50 m × 530 μm × 0 μm) was maintained at 50 °C for 3 min. The temperatures for sample entrance and the hydrogen flame ionization detector (FID) were 200 °C and 300 °C, respectively. The peak area in the chromatogram was utilized to calculate ethylene concentration by comparison to the standard curve. Three biological replicates were included for each treatment.

### 4.11. In Vivo Protein Degradation and Western Blotting

NbSAMS1 protein degradation by the 26S proteasome was tested in vivo using A. tumefaciens-mediated transient expression in *N. benthamiana* leaves. At 48 hpa, 100 mM MG132 (Aladdin Industrial Corporation, Shanghai, China) or 0.5% (*v*/*v*) DMSO was infiltrated into *N. benthamiana* leaves, and total protein was extracted for Western blot at 0 h, 4 h, and 8 h.

### 4.12. Computational Prediction of Protein–Protein Interactions

First, protein sequences of NbCBP1, NbSAMS1, and E3 ligase were downloaded from the *N. benthamiana* genome database (https://solgenomics.net/organism/Nicotiana_benthamiana/genome (accessed on 16 June 2024)). These protein structure were predicted using AlphaFold3 (https://alphafold.ebi.ac.uk/ (accessed on 18 June 2024)) and protein–protein docking simulations were performed using default parameters. The binding interfaces between NbCBP1, NbSAMS1, and E3 ligase were examined, and binding affinity was calculated to evaluate the possibility of direct interactions using PDBePISA online server (https://www.ebi.ac.uk/msd-srv/prot_int/ (accessed on 24 June 2024)). Finally, the predicted complex models were visualized and analyzed using PyMOL (version 3.0) and Chimera software (version 1.17.1).

### 4.13. Statistical Analysis and Graphical Presentaion

All data were analyzed using one-way ANOVA and Student’s *t*-tests in Prism version 8 (GraphPad, San Diego, CA, USA). The data are presented as mean ± standard deviation (SD) and analyzed using Student’s *t*-test. Asterisks indicate statistical significance (** *p* < 0.01; * *p* < 0.05; ns = not significant), and different letters indicate significant differences (*p* < 0.05).

## Data Availability

Data are contained within the article and Supplementary Materials.

## Supplementary Materials

The following supporting information can be downloaded at: https://www.mdpi.com/article/10.3390/ijms26031384/s1.
