# Supplementary material for: The NbCBP1-NbSAMS1 Module Promotes Ethylene Accumulation to Enhance Nicotiana benthamiana Resistance to Phytophthora parasitica Under High Potassium Status
Source: Int J Mol Sci. 2025 Feb 6;26(3):1384. doi: 10.3390/ijms26031384 (PMC11818782; doi:10.3390/ijms26031384)
Supplement: Supplementary file 1 [file ijms-26-01384-s001.zip › Figure_S2_SuppInfo.pdf]

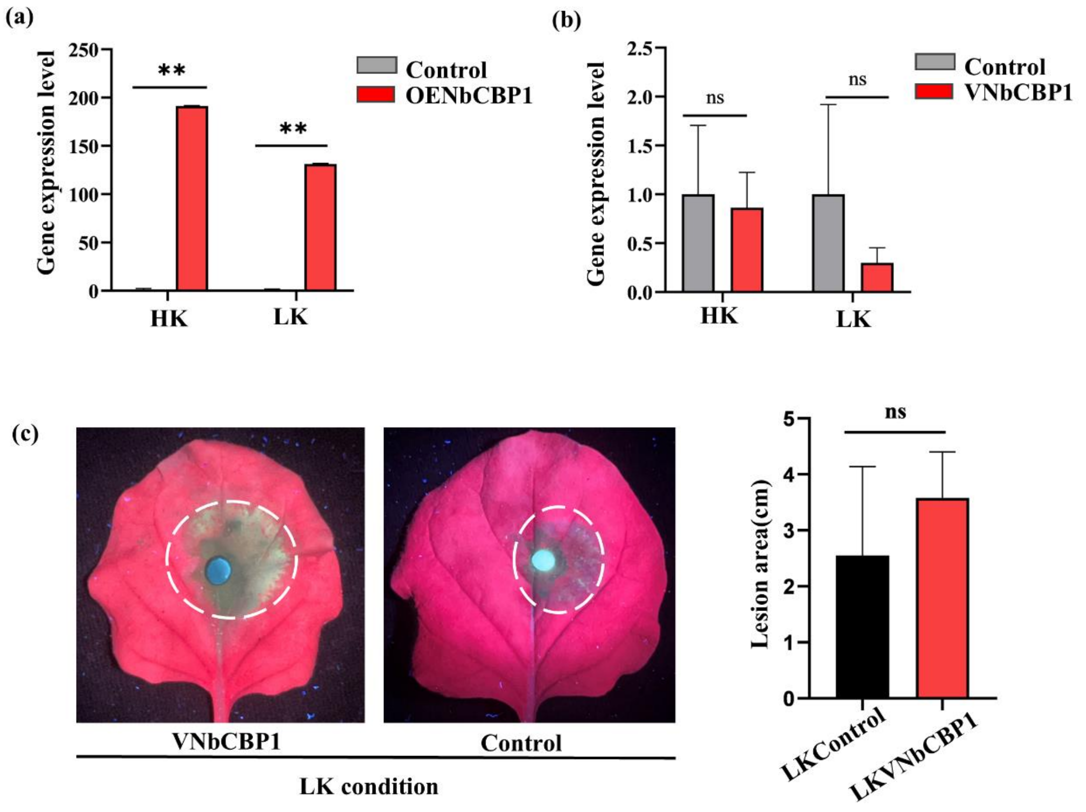

Figure S2: Verification of *NbCBP1* overexpression and silencing. RT-qPCR displays the (a) overexpression levels and (b) silencing efficiency of *NbCBP1* in HK and LK *N. benthamiana* inoculated with *P. parasitica. (c)* Lesion diameter analyses of silenced *NbCBP1* in LK *N. benthamiana.*
