# Supplementary material for: The NbCBP1-NbSAMS1 Module Promotes Ethylene Accumulation to Enhance Nicotiana benthamiana Resistance to Phytophthora parasitica Under High Potassium Status
Source: Int J Mol Sci. 2025 Feb 6;26(3):1384. doi: 10.3390/ijms26031384 (PMC11818782; doi:10.3390/ijms26031384)
Supplement: Supplementary file 1 [file ijms-26-01384-s001.zip › Figure_S3_SuppInfo.pdf]

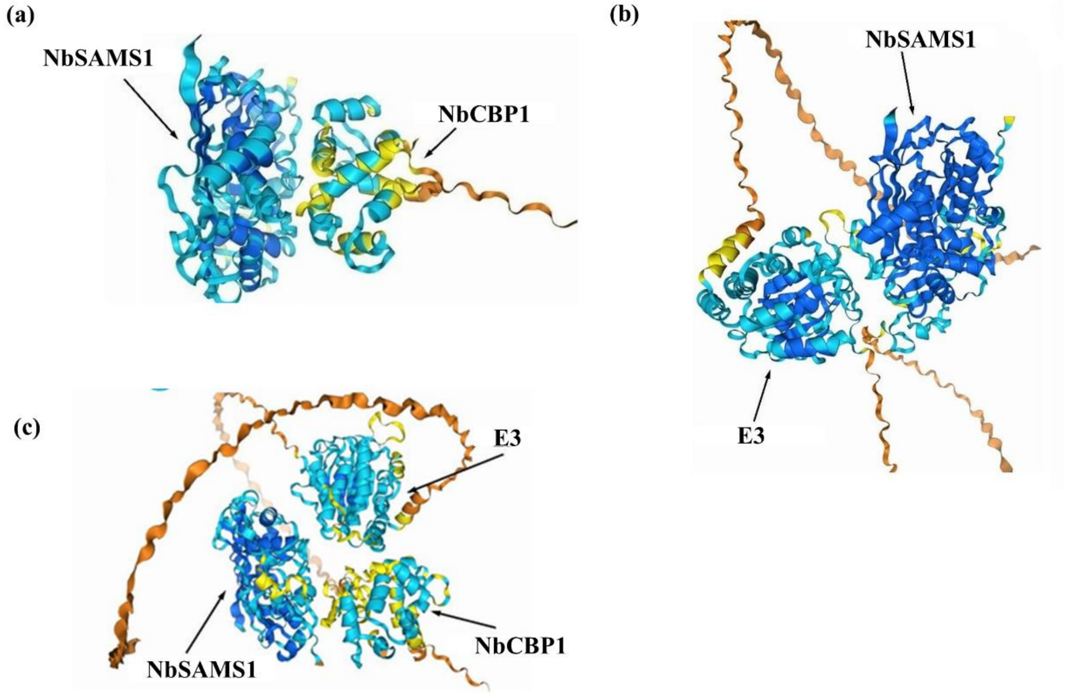


**Figure S3: AlphaFold Model predicted the potential interactions among NbCBP1, the E3 ligase, and NbSAMS1** (a)NbSAMS1 and NbCBP1 interaction (b)NbSAMS1 and E3 interaction. (c)NbSAMS1, NbCBP1 and E3 ligase
